# Expression profiling of white sponge nevus by RNA sequencing revealed pathological pathways

**DOI:** 10.1186/s13023-015-0285-y

**Published:** 2015-06-11

**Authors:** Wenping Cai, Beizhan Jiang, Tienan Feng, Jinfeng Xue, Jianhua Yang, Zhenghu Chen, Junjun Liu, Rongbin Wei, Shouliang Zhao, Xiaoping Wang, Shangfeng Liu

**Affiliations:** Department of Stomatology, Huashan Hospital, Fudan University, Shanghai, 200040 P. R. China; Laboratory of Oral Biomedical Science and Translational Medicine, School of Stomatology, Tongji University, Shanghai, 200072 P. R. China; School of Life sciences and Technology, Tongji University, Shanghai, 200065 P. R. China; State Key Laboratory of Medical Genetics, Central South University, Changsha, 410078 P. R. China; Department of Ophthalmology, Shanghai Tenth People’s Hospital, Tongji University School of Medicine, Shanghai, 200072 P. R. China

**Keywords:** White sponge nevus, Keratin7, Keratin13, Ubiquitin C (UBC), RNA sequencing (RNA-seq)

## Abstract

**Background:**

White sponge nevus (WSN) is a rare periodontal hereditary disease. To date, almost all WSN studies have focused on case reports or mutation reports. Thus, the mechanism behind WSN is still unclear. We investigated the pathogenesis of WSN using expression profiling.

**Methods:**

Sequence analysis of samples from a WSN Chinese family revealed a mutation (332 T > C) in the *KRT*13 gene that resulted in the amino acid change Leu111Pro. The pathological pathway behind the WSN expression profile was investigated by RNA sequencing (RNA-seq).

**Results:**

Construction of a heatmap revealed 24 activated genes and 57 reduced genes in the WSN patients. The ribosome structure was damaged in the WSN patients. Moreover, the translation rate was limited in the WSN patients, whereas ubiquitin-mediated proteolysis was enhanced.

**Conclusions:**

Our results suggest that the abnormal degradation of the KRT13 protein in WSN patients may be associated with keratin 7 (KRT7) and an abnormal ubiquitination process.

**Electronic supplementary material:**

The online version of this article (doi:10.1186/s13023-015-0285-y) contains supplementary material, which is available to authorized users.

## Background

White sponge nevus (WSN) is a rare periodontal hereditary disease that was first described by Hyde [1] and coined by Cannon [2]. It is characterized by white, thickened, folded and spongy lesions of the oral mucosa, although the esophageal, laryngeal, nasal and anogenital mucosa might also be affected [3]. Recently, KRT4 [4] and KRT13 [5] gene mutations were shown be the underlying cause of WSN. WSN is an autosomal dominant genetic disease in the oral mucosa. Although WSN patients do not experience significant physical pain, they often complain of an altered texture of the mucosa or changes in their physical appearance induced by the lesions. To develop better therapeutic strategies for WSN, it is important to understand the consequences of the associated genetic mutations and molecular changes. To date, many WSN cases have been reported around the world, including China [6–8], Italy [9–11], Japan [12, 13], the UK [14, 15], Spain [16], Scotland [17], Iran [18], Brazil [19], and Turkey [20]. However, the exact pathogenic mechanism behind WSN remains unclear. With the advent of next-generation sequencing technologies, RNA-seq has become a useful tool for defining the transcriptomes of cells. Moreover, this technology may be useful for analyzing gene expression at the exon level and delineating novel splicing variants [21–25]. Early applications of RNA-seq included expression profiling of yeast [26], mouse brains, liver tissues, skeletal muscle tissues [27], human embryonic kidneys, B cell lines [28] and early embryos [29]. RNA-seq offers several advantages over other expression profiling technologies, including higher sensitivity and the ability to detect splicing isoforms and somatic mutations [30]. To date, an RNA-seq analysis of WSN has not been published. Therefore, we applied RNA-seq technology to analyze WSN.

Recently, there have been many reports of new cases and mutations. However, the use of the modularity of transcriptional networks as a principle approach to understand this complex pathway has not been adequately explored. Elucidating the molecular mechanisms is a key factor for the development of successful treatments for WSN. In this study, we investigated the pathological pathway behind the WSN expression profile using RNA sequencing (RNA-seq).

This study provided a new direction for investigations into the mechanisms behind WSN, prenatal diagnosis and clinical treatment. Furthermore, our results provide a frame of reference and instructions for treating periodontal disease and other keratin-related diseases.

## Methods

### Ethics approval

Oral epidermis tissues were obtained from patients who were well informed of all of the purposes that the tissues might be used for in this study. This study was approved by Fudan University’s ethics committee.

### Clinical report

The proband in the WSN family was a 44-year-old male Chinese patient from Hunan province who was affected by white asymptomatic oral plaques that were clinically diagnosed as WSN. In this six-generation-family from Hunan province, there were 120 members. Using pedigree analysis, we determined that the genetic modes of the disease for 28 WSN patients from this family were autosomal dominant disorders. The incidence of WSN was 23.3 % in this family. The major lesionsin these patients were white plaques of the tongue and the buccal mucosa on both sides. The diagnosis of WSN was supported by the family history and the clinical and histopathological findings.

### Establishment of the cell lines

Oral epithelial cells from the normal subjects and the WSN patients were cultured in Dulbecco's modified Eagle's medium (DMEM) supplemented with 10 % fetal bovine serum (FBS), 100 units/ml penicillin, and 100 μg/ml streptomycin at 37 °C in 5 % CO_2_.

### RNA isolation and library construction

Total cellular RNA from the normal subjects and the WSN patients was extracted using the Trizol Reagent (Invitrogen, USA). Library construction was performed following the Illumina manufacturer’s suggestions. The libraries were sequenced on the Illumina Hiseq 2000 platform. Sequencing reads that contained polyA, low quality, and adapters were pre-filtered prior to mapping. The filtered reads were mapped on to the hg19 genome and the mm9 genome using default parameters with BWA aligner29; reads that failed to map to the genome were re-mapped to their respective mRNA sequences to capture reads that spanned exons.

### RNA-Seq analysis and pathogenic pathway analysis

We generated signal networks to identify and visualize the hub genes. We expected that module genes would have significant positive module membership values. Functional annotation was performed with the Database for Annotation, Visualization and Integrated Discovery (DAVID) Bioinformatics Resource. We focused our bioinformatics analysis on Ingenuity Pathways Analysis (IPA), Kyoto Encyclopedia of Genes and Genomes (KEGG) and Gene Set Enrichment Analysis (GSEA). RNA-seq represented an advanced method to investigate disease pathogenesis.

## Results

### Clinical report and establishment of the cell line

**Fig. 1:**
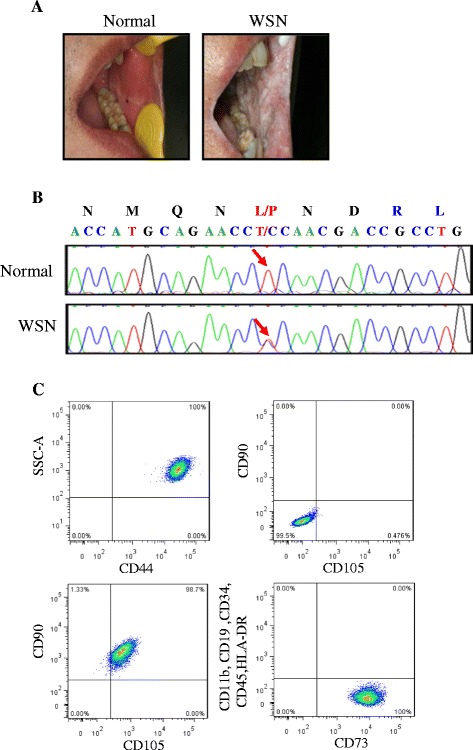
Mutation analysis and establishment of cell lines. **a**: White spongy oral plaques in the buccal mucosa of the normal subjects and the WSN patients; **b**: Partial DNA sequences of exon 1A of the KRT13 gene from a Chinese family. The arrow indicates the position of the mutation 332 T > C. This mutation predicts the amino acid change L111P in the KRT13 polypeptide from the WSN patient; **c**: FACS analysis of human mesenchymal stem cells (MSC) isolated from gum tissues

During the oral clinical examination, the WSN patient had white lesions located bilaterally on the lips, the lateral margin of the tongue and the bilateral buccal mucosa (Fig. 1a). The arrow in Fig. 1b indicates the position of the 332 T > C mutation identifiedin this family, this mutation predicts the amino acid change Leu111Pro. To investigate the pathogenic mechanism behind WSN, we collected tissues from the oral mucosa of WSN patients and normal controls from the same family for cell culture. The cells of the two cell lines attached and showed typical morphology for oral mucosa cells. Then, we established a stable mesenchymal stem cell line (MSC) from the subjects’ gum tissues and analyzed the cells by FACS (Fig. 1c).

### RNA-Seq analysis and pathogenic pathway analysis

**Fig. 2:**
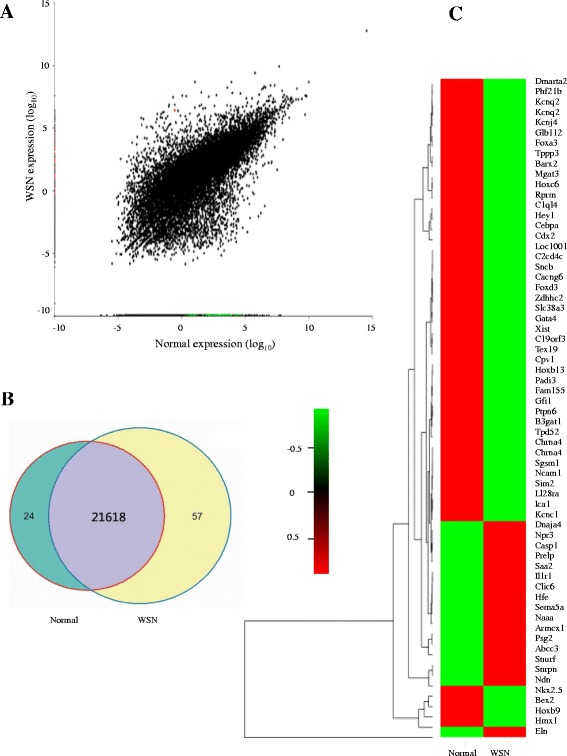
RNA-seq analysis. **a**: The distribution of differentially expressed genes between the normal subjects and the WSN patients; **b**: Scatterplot showing the number of activated (red) and reduced (green) genes in the normal subjects compared to the WSN patients; **c**: Heatmap showing the relative expression of activated genes in the WSN patients (n = 24)

**Fig. 3:**
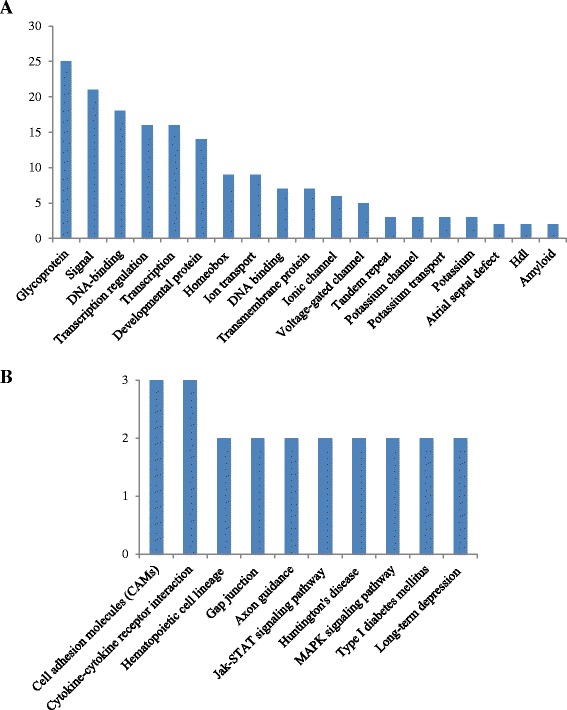
Functional analysis and pathway analysis. **a**: Functional annotation of differentially expressed genes; **b**: Pathway analysis

**Fig. 4:**
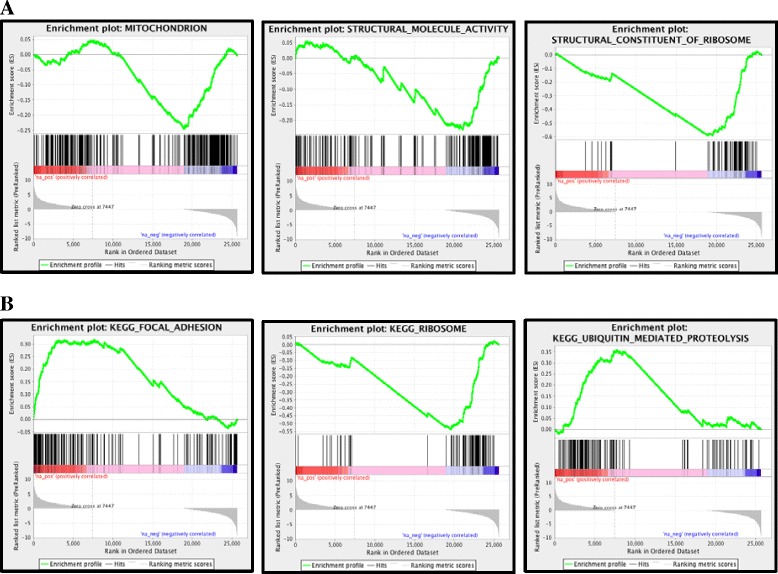
Gene Ontology and KEGG Enrichment analysis. **a**: The strongest enriched Gene Ontology terms in the GSEA/GO analysis; **b**: The snapshot of KEGG enrichment

The scatterplot in Fig. 2a depicts the number of activated (red) and reduced (green) genes in the normal subjects compared to the WSN subjects. The heatmap demonstrated the presence of 24 activated genes and 57 reduced genes in the WSN patient relative to the control (Fig. 2b). Using the IPA software package, we identified 19 significant bio-function terms and 10 significant canonical pathways through KEGG analysis (Fig. 3a and b) (Additional files 1 and 2).The strongest enriched gene ontology terms found in the GSEA/GO analysis indicated that genes involved in the structural constituents of ribosomes are expressed at reduced levels in WSN patients; this finding is in agreement with the observation that the structure of the ribosome is damaged in WSN patients (Fig. 4a). The snapshot of the KEGG enrichment pathways showed that protein degradation levels and ubiquitin-mediated proteolysis were enhanced in the WSN patients (Fig. 4b). Next, we chose the top 20 upregulated classes between the normal subjects and the WSN patients. There were significant differences between the size, enrichment score (ES) and NES (Tables 1 and 2) between the 2 groups. The WSN enrichment area was mainly focused on monooxygenase activity, the JAK Stat cascade, and extracellular regions. In contrast, enrichment areas from the normal patient included the detection of stimuli and G-protein signaling.

**Table 1:** GSEA analysis of a normal subject

Number	Upregulated on normal	Size	ES	NES
1	DETECTION_OF_STIMULUS	27	−0.6574245	−1.8061078
2	G_PROTEIN_SIGNALING_COUPLED_TO_IP3_SECOND_MESSENGERPHOSPHOLIPASE_C_ACTIVATING	24	−0.6308761	−1.6817592
3	PHOSPHOINOSITIDE_MEDIATED_SIGNALING	27	−0.60555935	−1.6654842
4	POTASSIUM_CHANNEL_ACTIVITY	32	−0.56262994	−1.5977739
5	HYDROLASE_ACTIVITY_ACTING_ON_CARBON_NITROGEN_NOT_PEPTIDEBONDS	25	−0.5852488	−1.5726966
6	POTASSIUM_ION_TRANSPORT	36	−0.53710914	−1.5694768
7	CATION_CHANNEL_ACTIVITY	82	−0.46867418	−1.5630565
8	SUBSTRATE_SPECIFIC_CHANNEL_ACTIVITY	106	−0.44555083	−1.5522181
9	ANION_TRANSPORT	16	−0.6484232	−1.5312126
10	GATED_CHANNEL_ACTIVITY	86	−0.44908124	−1.5255814
11	HORMONE_METABOLIC_PROCESS	18	−0.61389524	−1.5086162
12	SULFOTRANSFERASE_ACTIVITY	18	−0.60654145	−1.4967437
13	ION_CHANNEL_ACTIVITY	101	−0.43095177	−1.4898388
14	HEMATOPOIETIN_INTERFERON_CLASSD200_DOMAIN_CYTOKINE_RECEPTOR_BINDING	20	−0.5910758	−1.4891092
15	CELL_CYCLE_PROCESS	107	−0.41814068	−1.4650342
16	VOLTAGE_GATED_CATION_CHANNEL_ACTIVITY	44	−0.48482108	−1.463665
17	AXONOGENESIS	26	−0.54742056	−1.4614419
18	HEART_DEVELOPMENT	19	−0.5847264	−1.4440396
19	LIGAND_GATED_CHANNEL_ACTIVITY	30	−0.51191664	−1.4319984
20	NEUROTRANSMITTER_RECEPTOR_ACTIVITY	32	−0.500388	−1.4252495

**Table 2:** GSEA analysis of a WSN patient

Number	Upregulated on WSN	Size	ES	NES
1	MONOOXYGENASE_ACTIVITY	17	0.76	1.85
2	JAK_STAT_CASCADE	21	0.69	1.8
3	EXTRACELLULAR_REGION_PART	216	0.47	1.8
4	SUGAR_BINDING	22	0.69	1.79
5	CARBOHYDRATE_BINDING	45	0.59	1.79
6	LIPID_RAFT	15	0.73	1.75
7	PROTEASE_INHIBITOR_ACTIVITY	21	0.66	1.72
8	POSITIVE_REGULATION_OF_CYTOKINE_BIOSYNTHETIC_PROCESS	17	0.67	1.67
9	NEGATIVE_REGULATION_OF_MULTICELLULAR_ORGANISMAL_PROCESS	21	0.66	1.67
10	PROTEINACEOUS_EXTRACELLULAR_MATRIX	65	0.51	1.67
11	RAS_GTPASE_BINDING	17	0.68	1.66
12	EXTRACELLULAR_REGION	281	0.43	1.66
13	STRUCTURAL_CONSTITUENT_OF_MUSCLE	15	0.7	1.66
14	EXTRACELLULAR_MATRIX	66	0.51	1.65
15	COAGULATION	31	0.58	1.65
16	POSITIVE_REGULATION_OF_TRANSLATION	22	0.62	1.65
17	VIRAL_REPRODUCTIVE_PROCESS	22	0.62	1.64
18	EXTRACELLULAR_SPACE	155	0.44	1.63
19	BLOOD_COAGULATION	30	0.58	1.62
20	PROTEIN_TYROSINE_PHOSPHATASE_ACTIVITY	29	0.59	1.62

We used signaling networks to identify and visualize the hub genes. We expected the module genes to have significant positive module membership values. Then, we constructed a network using published data sets and generated an independent list of hub genes.

**Fig. 5:**
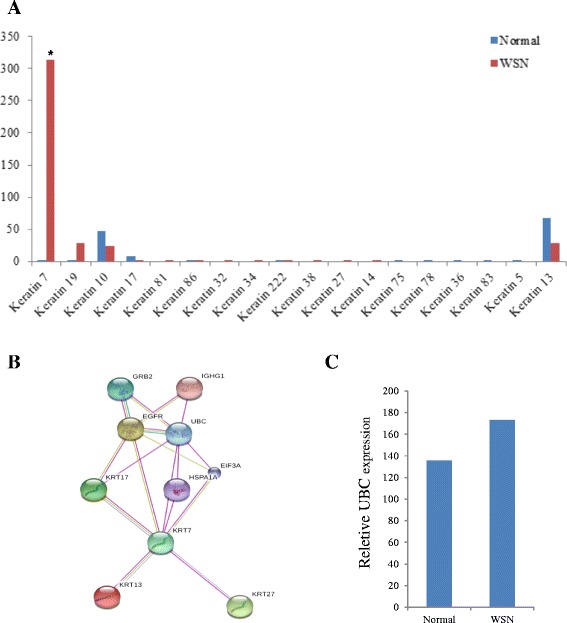
Pathogenic mechanism analysis. **a**: The KRT expression levels between the normal subjects and the WSN patients; **b**: Module visualization of network connections and associated functions. Bioinformatics analysis of target genes and network analysis of these genes using the String 8.3 software indicated the central involvement of KRT 13 signaling; **c**: The UBC expression levels between the normal subjects and the WSN patients

We compared the expression levels of different KRT proteins from the normal subjects and the WSN patients. The KRT7 expression level was lower in the WSN patients; however, there was almost no change in the KRT13 levels (Fig. 5a).The constructed pathway was in agreement with the module visualization of network connections and associated functions. The function of KRT13 was dependent on KRT7 and UBC (Fig. 5b). There was almost no changein the UBC expression levels between the normal subjects and the WSN samples (Fig. 5c). Thus, the abnormal degradation of the KRT13 protein in the WSN patients may be associated with an abnormal ubiquitination process.

## Discussion

With the development of sequencing technology, there are two important bioinformatic gene expression profiling methods: Microarray and RNA-Seq. In oral research, microarrays have diverse applications in oral cancers, including early diagnosis of the transformation of premalignant lesions, identification of malignancy in tissue biopsies, drug discovery, identification of biomarkers, and subclassification of histologically identified tumors [31]. In premalignant lesions, such as leukoplakias and erythroplakias, microarrays have been used to identify genes that could serve as biomarkers for dysplastic lesions with the potential to progress to cancer [32]. For clinical applications, microarrays have been used for a longer period of time and will probably have regulatory approvals for diagnostic use prior to RNA-Seq obtaining approvals. RNA-Seq will eventually be used more routinely than microarray, but right now the techniques can be complementary to each other. Microarrays will not become obsolete but might be relegated to only a few uses. RNA-Seq clearly has a bright future in bioinformatic data collection [33].

WSN is a rare oral hereditary disease. Gene expression profiling of WSN patientshas not elucidated the mechanisms behind this disease. In this study, we analyzed the expression differences at the gene level between normal subjects and WSN patients and found 81 differentially expressed genes. These genes was divided into 8 categories according to their gene function and 10 canonical pathways, which provided important clues for understanding the molecular mechanisms behind WSN pathogenesis.

In summary, we demonstrated that the use of RNA-seq markedly improved the transcriptome quantification associated with WSN. We expect that RNA-seq will also be useful for quantitatively delineating the structures, isoforms, and specific expression patterns of both coding genes and non-coding regulatory RNAs. Furthermore, RNA-seq analysis has the advantage of providing quantitative results, which is in contrastto exome or genomic sequencing.

To the best of our knowledge, WSN is an autosomal dominant genetic disease. Therefore, there is no appropriate animal model or no effective therapeutic drugs have been developed. Although WSN patients do not exhibit significant physical pain, they often complain of an altered texture of the mucosa or changes to their physical appearance induced by the lesions. To develop better therapeutic strategies for WSN, it is important to understand the consequences of the associated genetic mutations and molecular changes. Therefore, we investigated the pathogenesis and signaling pathways involved in WSN using RNA-seq. Our results suggested that the KRT13 mutation may be associate with KRT7 and UBC. UBC is a protein-coding gene that encodes a ubiquitin protein that exists either covalently attached to another protein or free (unanchored). Our previous study found that the abnormal degradation of the KRT13 protein in WSN patients contributed to an abnormal ubiquitination process [6]. Therefore, a key target for the treatment of WSN is to prevent the degradation of the KRT13 protein.

Our study utilized a next generation sequencing platform to comprehensively characterize the KRT13-related WSN transcriptome for the first time. It provided the basis for an understanding of the molecular mechanisms behind WSN pathogenesis at a system-wide level. Future research based on our findings may speed up the discovery of novel biomarkers and drug targets that can be used to improve the diagnosis and therapy of WSN. The results of our RNA-Seq analysis suggested that the abnormal degradation of the KRT13 protein in WSN patients may be associated with keratin 7 (KRT7) and an abnormal ubiquitination process. The structure of the ribosome was found to be damaged in WSN patients. Moreover, the translation rate was reduced in WSN patients, whereas ubiquitin-mediated proteolysis was enhanced. Therefore, the development of a valuable drug to reduce the degradation of KRT13is crucial for WSN patients. We are optimistic that the problem will be solved with future studies on WSN. We also expect the development of RNA-Seq to enable applications involved in determining the structural dynamics of the transcriptome and the pathogenic mechanism of disease.

Although WSN patients had no physical pain significantly, but they often complained of an altered texture of the mucosa or the bad looking created by the lesions. Many WSN patients made therapy treatments medication with nystatin, antihistamines, vitamins and mouth rinses. Azithromycin, tetracycline [5], chlorhexidine [34], Victoria A acid [35] and penicillin had succeed in the clinical progress. However, there is no standard treatment protocol for WSN till now. We applied RNA-seq technology to explor the WSN mechanism. The Human induced pluripotent stem cells (iPSCs) represent an excellent tool for many clinical trials [36]. The RNA-seq technology and induced pluripotent stem cells (iPSCs) technology may be a point way to the treatment of the rare disease. Meanwhile the WSN patient should perform a careful oral hygiene to reduce infection in the oral cavity. To lead to the proper diagnosis and treatment of this rare disease, it is great importance of collaboration between anamnesis, clinical examination and pathologic findings. With the further research of WSN, there is optimism that the problem will be solved in the next decade years.

## Conclusions

The genetic disease WSN occurs infrequently. Experts have struggled to grasp factors contributing to its clinical symptoms, gene mutations and treatment; however, the mechanism behind the disease is still unclear. Here, we report the pathogenic mechanism of WSN. Our results suggest that the abnormal degradation of the KRT13 protein in WSN patients may be associated with KRT7 and an abnormal ubiquitination process. This finding may contribute to the development of a molecular therapy for WSN. Gene-based diagnosis and therapy for WSN patients may become available in the near future and may provide references and instructions for treating other keratin-associated diseases. This finding will hopefully improve the levels of prenatal diagnoses and treatment of rare diseases.

## Additional files

Additional file 1: Table S1. Functional annotation of different expression genes. Additional file 2: Table S2. Pathway analysis.

## Abbreviations

KRT: Keratin
KRTs: Keratins
KRT4: Keratin 4
KRT13: Keratin 13
KRT7: Keratin 7
RNA-seq: RNA sequencing
WSN: White sponge nevus
DAVID: Database for Annotation Visualization and Integrated Discovery
IPA: Ingenuity Pathways Analysis
KEGG: Kyoto Encyclopedia of Genes and Genomes
GSEA: Gene Set Enrichment Analysis
iPSCs: induced Pluripotent Stem Cells.
