# Erratum to: Expression profiling of white sponge nevus by RNA sequencing revealed pathological pathways

**DOI:** 10.1186/s13023-015-0308-8

**Published:** 2015-09-23

**Authors:** Wenping Cai, Beizhan Jiang, Tienan Feng, Jinfeng Xue, Jianhua Yang, Zhenghu Chen, Junjun Liu, Rongbin Wei, Shouliang Zhao, Xiaoping Wang, Shangfeng Liu

**Affiliations:** Department of Stomatology, Huashan Hospital, Fudan University, Shanghai, 200040 P. R. China; Laboratory of Oral Biomedical Science and Translational Medicine, School of Stomatology, Tongji University, Shanghai, 200072 P. R. China; School of Life sciences and Technology, Tongji University, Shanghai, 200065 P. R. China; State Key Laboratory of Medical Genetics, Central South University, Changsha, 410078 P. R. China; Department of Ophthalmology, Shanghai Tenth People’s Hospital, Tongji University School of Medicine, Shanghai, 200072 P. R. China

## Erratum

After publication of [1] the authors realised that the original publication had two descriptive errors when referring to diseases. “periodontal” was written rather than “mucous membrane” in the article on page 1, lines 12 and 25. “periodontal disease” was written rather than “mucous membrane hyperkeratosis” in the article on page 2, lines 72 and 73. The correct spelling of these is included in this erratum.
