# Supplementary material for: Expression profiling of white sponge nevus by RNA sequencing revealed pathological pathways
Source: Orphanet J Rare Dis. 2015 Jun 11;10:72. doi: 10.1186/s13023-015-0285-y (PMC4474461; doi:10.1186/s13023-015-0285-y)
Supplement: Additional file 1: Table S1. — Functional annotation of different expression genes. [file 13023_2015_285_MOESM1_ESM.pptx]

## Slide 1
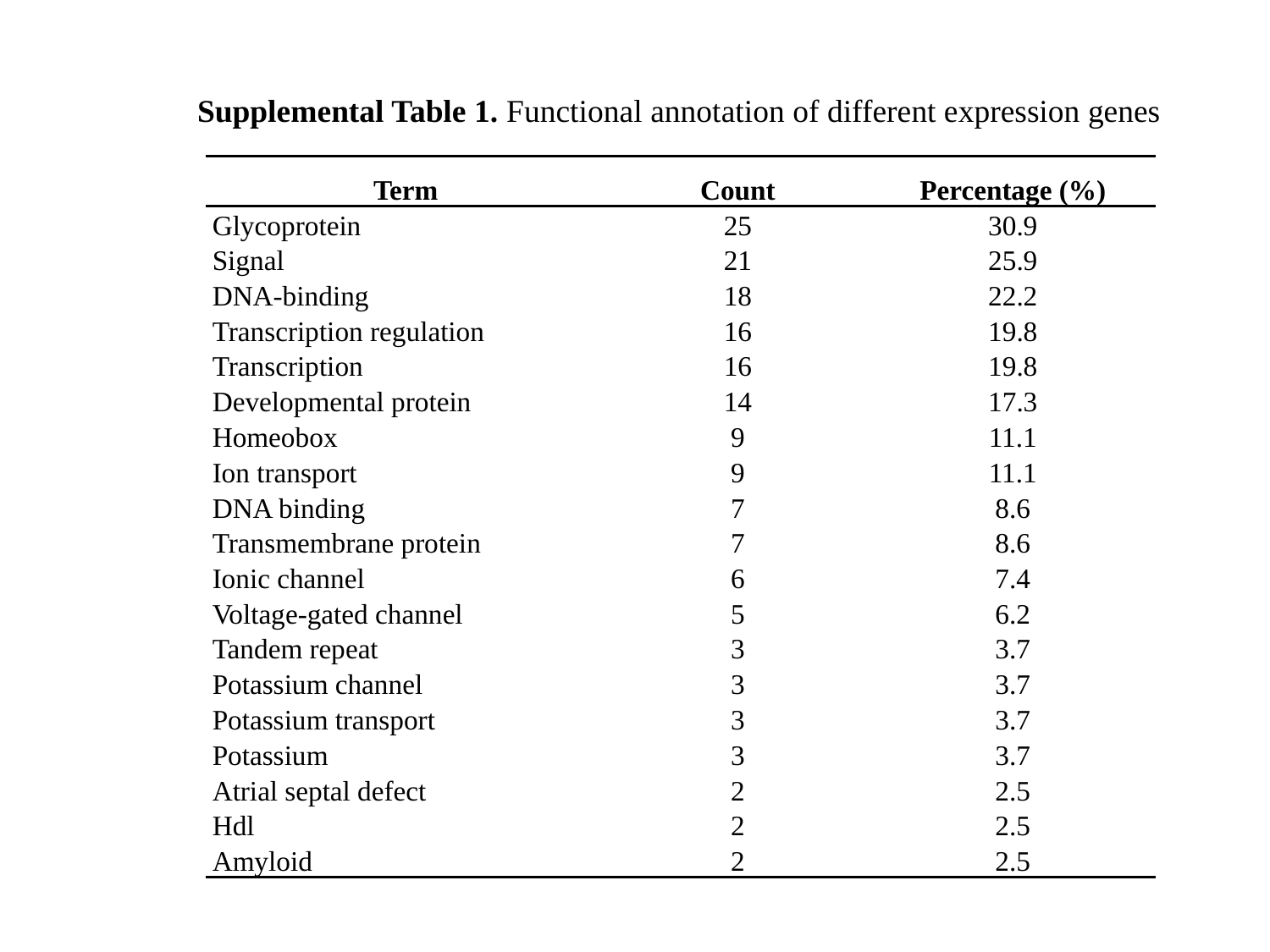

Supplemental Table 1. Functional annotation of different expression genes
| Term | Count | Percentage (%) |
| --- | --- | --- |
| Glycoprotein | 25 | 30.9 |
| Signal | 21 | 25.9 |
| DNA-binding | 18 | 22.2 |
| Transcription regulation | 16 | 19.8 |
| Transcription | 16 | 19.8 |
| Developmental protein | 14 | 17.3 |
| Homeobox | 9 | 11.1 |
| Ion transport | 9 | 11.1 |
| DNA binding | 7 | 8.6 |
| Transmembrane protein | 7 | 8.6 |
| Ionic channel | 6 | 7.4 |
| Voltage-gated channel | 5 | 6.2 |
| Tandem repeat | 3 | 3.7 |
| Potassium channel | 3 | 3.7 |
| Potassium transport | 3 | 3.7 |
| Potassium | 3 | 3.7 |
| Atrial septal defect | 2 | 2.5 |
| Hdl | 2 | 2.5 |
| Amyloid | 2 | 2.5 |

## Slide 2
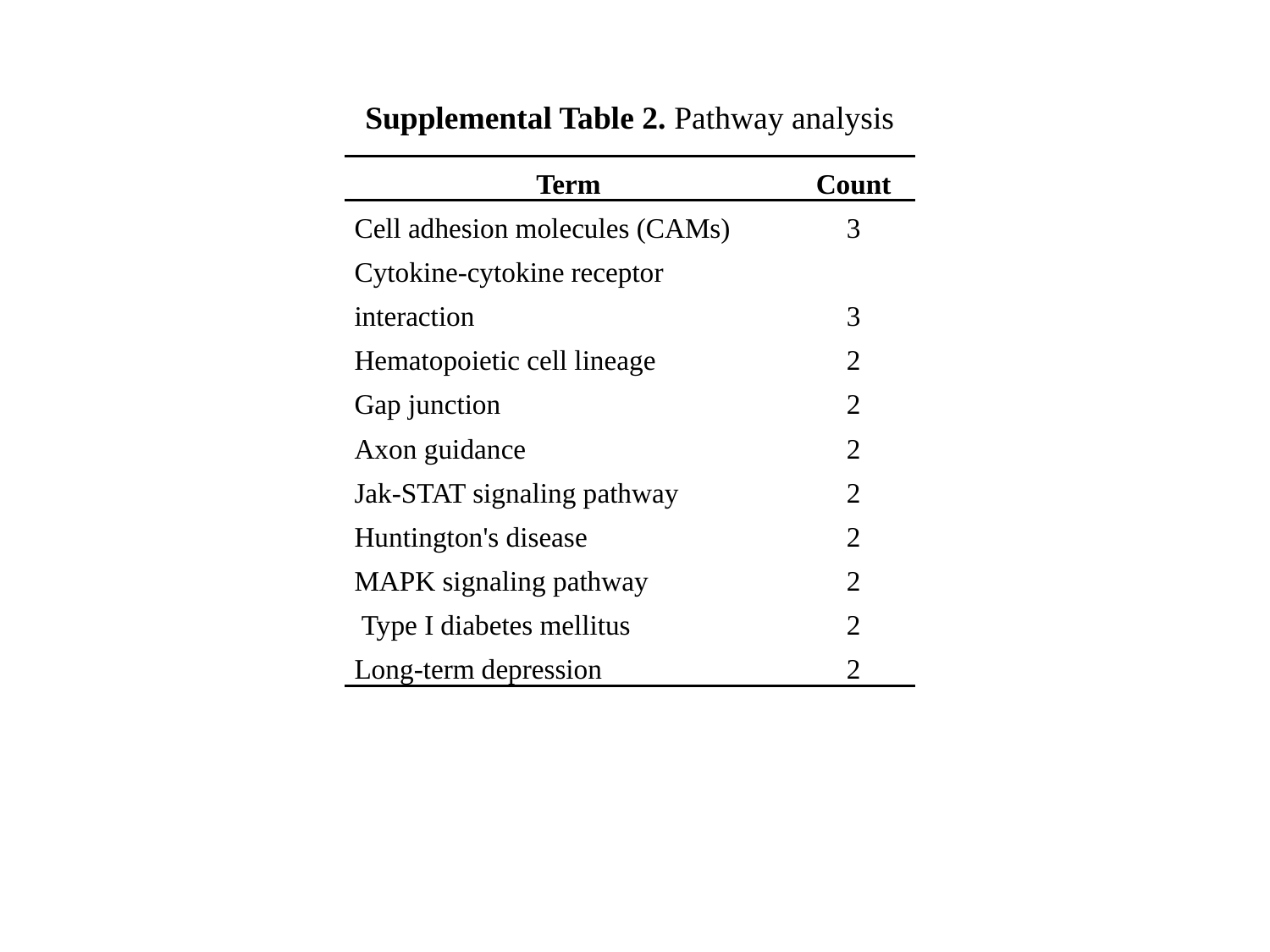

Supplemental Table 2. Pathway analysis
| Term | Count |
| --- | --- |
| Cell adhesion molecules (CAMs) | 3 |
| Cytokine-cytokine receptor interaction | 3 |
| Hematopoietic cell lineage | 2 |
| Gap junction | 2 |
| Axon guidance | 2 |
| Jak-STAT signaling pathway | 2 |
| Huntington's disease | 2 |
| MAPK signaling pathway | 2 |
| Type I diabetes mellitus | 2 |
| Long-term depression | 2 |
