# Supplementary material for: Expression profiling of white sponge nevus by RNA sequencing revealed pathological pathways
Source: Orphanet J Rare Dis. 2015 Jun 11;10:72. doi: 10.1186/s13023-015-0285-y (PMC4474461; doi:10.1186/s13023-015-0285-y)
Supplement: Additional file 2: Table S2. — Pathway analysis. [file 13023_2015_285_MOESM2_ESM.pdf]

WILEY

Wiley Editing Services

LANGUAGE EDITING

CERTIFICATE

This document certifies that the manuscript listed below was edited for proper English language, grammar, punctuation, spelling, and overall style by one or more of the highly qualified native English speaking editors at Wiley Editing Services.

Manuscript title:

Expression profiling of white sponge nevus by RNA sequencing revealed pathological pathways

Authors:

Wenping Cai, Beizhan Jiang, Jinfeng Xue, Tienan Feng, Jianhua Yang, Zhenghu Chen, Junjun Liu, Rongbin Wei, Shouliang Zhao, Xiaoping Wang, Shangfeng Liu

Date Issued:

March 5, 2015

Certificate Verification Key:

6D4D-B645-627F-1AE8-22F9

This certificate may be verified at https://secure.wileyeditingservices.com/certificate. This document certifies that the manuscript listed above was edited for proper English language, grammar, punctuation, spelling, and overall style. Neither the research content nor the authors' intentions were altered in any way during the editing process. Documents receiving this certification should be English-ready for publication; however, the author has the ability to accept or reject our suggestions and changes. If you have any questions or concerns about this document or certification, please contact help@wileyeditingservices.com.

CERTIFIED Wiley Editing Services

Wiley Publishing Services is a service of Wiley Publishing. Wiley's Scientific, Technical, Medical, and Scholarly (STMS) business serves the world's research and scholarly communities, and is the largest publisher for professional and scholarly societies. Wiley is committed to providing high quality services for researchers. To find out more about Wiley Editing Services, visit wileyeditingservices.com. To learn more about our other author services provided by Wiley Publishing, visit authorservices.wiley.com.
